# Risk Factors and Outcomes for Postoperative Ileus After Small Intestinal Fistula Excision in Patients With Diffuse Extensive Abdominal Adhesions

**DOI:** 10.3389/fsurg.2021.632241

**Published:** 2021-02-19

**Authors:** Weiliang Tian, Ming Yan, Xin Xu, Zheng Yao, Risheng Zhao

**Affiliations:** ^1^Department of General Surgery, Jinling Hospital, Nanjing, China; ^2^Department of General Surgery, Nanjing Jiangning Hospital, Nanjing, China

**Keywords:** postoperative ileus, abdominal surgery, gastrointestinal tract, adhesion, intestinal fistula, outcomes

## Abstract

**Purpose:** The study aimed to investigate the risk factors for postoperative ileus (POI) after small intestinal fistula excision (SIFE) in patients with diffuse extensive abdominal adhesions.

**Methods:** From October 2010 to December 2019, we enrolled patients who underwent SIFE and had diffuse extensive abdominal adhesions. Patients were divided into the POI group and the non-POI group according to its occurrence. We then investigated and analyzed the clinical characteristics of both groups.

**Result:** A total of 247 patients were enrolled into the study. There were 100 patients in the POI group, and 147 patients in the non-POI group. A multi-variable logistic regression analysis revealed that blood loss during SIFE (OR = 1.001; 95% CI: 1.000–1.259; *P* = 0.012), postoperative lactate(OR = 1.212; 95% CI: 1.001–1.304; *P* = 0.015), grade V abdominal adhesions (OR = 2.518; 95% CI: 1.814–3.44; *P* = 0.024), and time for recovery of lactate <2 mmol/L (OR = 2.079; 95% CI: 1.599–3.616; *P* = 0.026) were associated with POI. Moreover, POI was also associated with prolonged postoperative stay in the hospital (HR = 3.291; 95% CI: 2.511–4.172; *P* = 0.014).

**Conclusion:** Blood loss during operation, grade V abdominal adhesions, positive fluid balance within 48 h of operation, and time for recovery of lactate were the risk factors for POI after SIFE in patients with diffuse extensive abdominal adhesions.

## Introduction

Postoperative ileus (POI) is a common complication following abdominal surgery (1). Inhibitory gastrointestinal tract reflexes, local inflammatory responses, and pharmacologic interactions are all reported as factors triggering the development of POI (2–5).

Because of previous abdominal infection, patients with a small intestinal fistula always had abdominal adhesions during the definitive fistula excision (6). During the surgical procedure, abdominal adhesions led to a massive operation due to the stimulation of local inflammatory responses and the trigger of POI (7). Besides, during the small intestinal fistula excision (SIFE), the abdominal cavity might get contaminated due to the digestive overflow from the fistula or the damaged areas during the procedure of dissociation. The digestive overflow might also be a significant factor leading to local inflammatory responses and POI.

As a result, patients receiving a SIFE have a high risk of POI. Our study refers to a specific group of patients requiring attention, regarding the development of POI after SIFE. In the present study, we reviewed the medical characteristics of such patients receiving SIFE and investigated POI risk factors.

## Methods

This retrospective case-control study was performed at two tertiary hospitals. The institutional review board (IRB) provided the necessary approval.

### Patients Population and Grouping

From October 2010 to December 2019, we enrolled patients who underwent SIFE and had diffuse extensive abdominal adhesions. The exclusion criteria were: (1) patients younger than 18 years and (2) patients without a complete medical record.

Patients in our study were divided into the POI group and the non-POI group according to its occurrence. We then investigated and analyzed the clinical characteristics of the two groups.

### Status of Abdominal Adhesions

**Figure 1:**
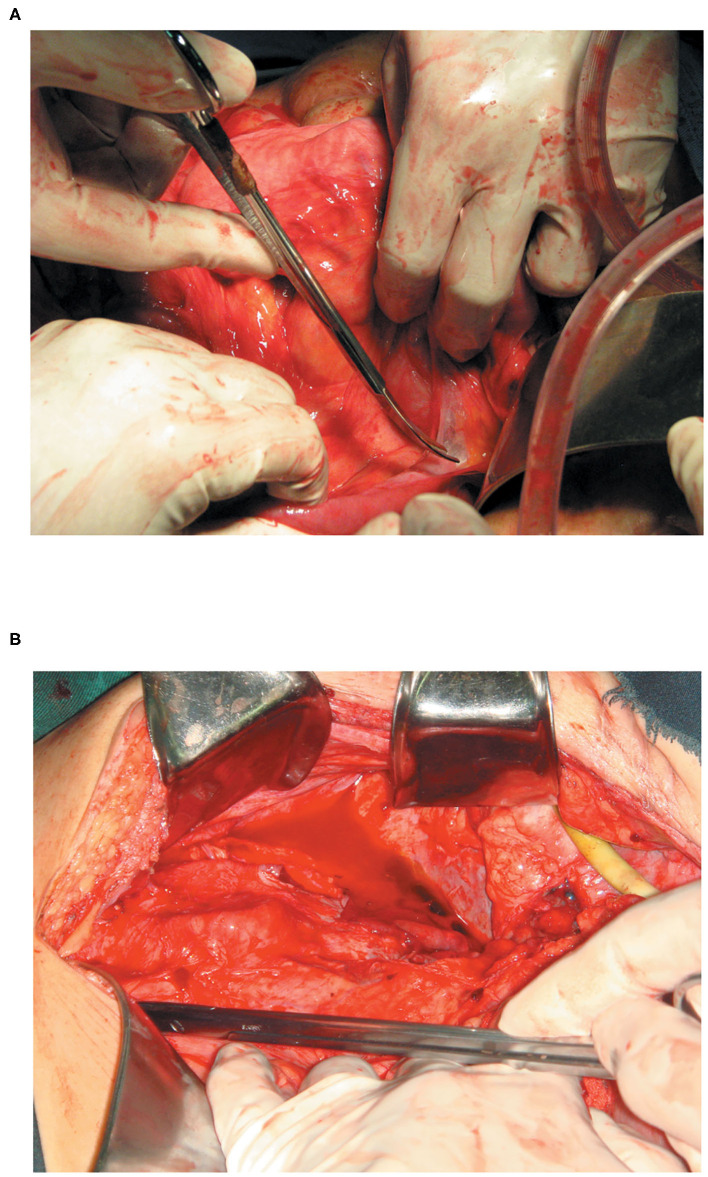
**(A)** With grade IV intestinal adhesions (diffuse extensive adhesions and easily lysed) more than 50% of the intestinal tract in the abdominal cavity was adherent, with poor dissociation. However, the shape of the intestine in the area could be identified, and could be easily lysed. **(B)** With grade V intestinal adhesions (diffuse extensive, dense adhesions, and difficult to lyse) more than 50% of the adhesions tissue between the two segments of the intestine was invaded by abundant capillaries, and the shape of the intestine in the area could not be identified.

We evaluated the status of abdominal adhesions according to the method of Hobson et al. during the SIFE (8). The abdominal adhesions were classified into the following five grades: Grade I = no adhesions; Grade II = minimal adhesions localized to one or two areas; Grade III =diffuse adhesions, but not extensive; Grade IV =diffuse extensive adhesions, easily lysed; Grade V = diffuse extensive, dense adhesions, difficult to lyse. Grade IV and V abdominal adhesions were defined as diffuse extensive abdominal adhesions (Figures 1A,B).

The definition of abdominal adhesions was further refined. Under “Diffuse extensive adhesions,” over 50% of the intestinal tract in the abdominal cavity was adherent, with poor dissociation. Under “Dense adhesions,” more than 50% of the adhesions tissue between the two segments of the intestine was invaded by abundant capillaries, and the shape of the intestine in the area could not be identified.

### Small Intestinal Fistula Excision

A SIFE was planned when patients met the following conditions: (1) blood biochemistry, blood routine, and infection index (CRP and PCT) were normal for at least more than 1 month; (2) patients had a BMI higher than 18.5 kg/m^2^ and had normal physical strength; (3) patients had no fever, abdominal distention, or vomiting for more than 1 month at least; (4) it had been more than 2 months since the last abdominal operation(for patients with open abdomen or enteroatmospheric fistula, the interval from the last operation to SIFE would be as long as 4 months, at least) (9). During the period preparing for the SIFE, adequate drainage and nutritional support were the primary treatments. Although we did not deliberately pursue the spontaneous closure of the fistula, it was achieved in some patients.

During the SIFE, the digestive tract (including the small intestine and the colon) was gradually dissociated. In the process, the length of the intestine and the grade of abdominal adhesions were evaluated. During the dissociation, small break areas on the intestine were sutured, and fistulas and large break areas were resected. During the resection, end-to-side anastomosis was performed using a curved intraluminal stapler (Ethicn, Endo-Surgery, NM, USA). Patients with an open abdomen or an enteroatmospheric fistula received an one-stage abdominal closure using a biological patch. In all cases, intra-intestinal splinting was performed before abdominal closure. During the process, the appendix was removed and a flexible tube was inserted into the small intestine through the stump retrogradely, for a sufficient length.

### Perioperative Care and POI

Total enteral nutrition [EN, Nutrison fiber (1.0 kcal/mL), Nutricia, Wuxi, China] support *via* a nasojejunal intestinal tube was given to each patient during the whole period of fistula treatment (from admission to discharge). The feeding rate was 80 mL/h, and the total energy supply was 1,500 kcal/day.

The preoperative preparation included the following: (1) normal bowel preparation; (2) placement of a nasogastric tube for decompression; and (3) use of antibiotics. A central venous catheter was placed in every patient's jugular vein after the induction of anesthesia. Patients with hemoglobin (Hb) <100 g/L and/or albumin (Alb) <30 g/L required red blood cell and/or human serum Alb administration during the postoperative treatment, respectively. Alb was the only colloid used in postoperative fluid management. Synthetic colloid was not used postoperatively. The postoperative fluid infusion was primarily based on central venous pressure (CVP), mean arterial pressure (MAP), heart rate (HR), and urine volume. After adequate fluid treatment, if the MAP was still <65 mmHg, vasoactive drugs (norepinephrine) were given.

On the 1st day after SIFE, parenteral nutrition (PN) was given. After the defecation, the total EN was gradually restored. If defecation was not normal and abdominal distention was present on the 7th day after EFE, POI was diagnosed.

### Data Collection and Analysis

The baseline preoperative data, including general patient information (gender, BMI, age, etc.), etiology, operation leading to fistula, fistula output, and ASA score, were collected 1 day before SIFE. The length of the small intestine and the grade of abdominal adhesions (10) were evaluated during the SIFE. Besides, the operation details such as operative duration, intraoperative hemorrhage, blood transfusion volume, and the presence of multiple anastomoses were collected during the SIFE. After the SIFE, a laboratory examination of blood was performed immediately. Then, postoperative lactate, leukocyte, Hb, and Alb were collected. We reviewed the input of Alb and RBC suspension within 48 h of SIFE. We investigated the time for recovery of lactate <2 mmol/L, Alb >35 g/L, and RBC >90 g/L.

Statistical analysis was performed using the Statistical Package for Social Science (SPSS) version 26.0 for Windows (IBM, Analytics, Armonk, NY). The student's *t*-test and a Mann–Whitney *U*-test were used to compare continuous variables. Fisher's exact test was used to compare categorical variables. A multivariate logistic regression analysis was used to evaluate the risk factors for POI. The multivariate cox-regression analysis and the K–M survival analysis were used to investigate patients' outcomes with POI. A *P*-value of <0.05 was considered statistically significant.

## Results

### Population

From October 2010 to December 2019, a total of 598 patients received a SIFE. Of these patients, 266 had diffuse extensive abdominal adhesions (grade IV or V). Of these 266 patients, 19 were excluded (15 patients without a completed medical record and four patients younger than 18 years), and 247 were enrolled in our study.

Of the 247 patients, 118 were females (47.78%). The mean age of the 247 patients was 39.97 ± 10.09 years. The median of postoperative BMI was 19.5 (19–22.75) kg/m^2^. The fistula resulted from trauma in 148 patients (59.91%), from mechanical ileus in 86 patients (34.82%), and mesenteric thrombosis in 13 patients (5.26%). Of the 247 patients, 60 (24.29%) had received an intestinal repair, and the rest 187 (75.71%) had received intestinal excision and anastomosis, which led to an intestinal fistula. One hundred of the 247 patients with a POI were classified under the POI group, and the incidence of POI was 40.49%. The remaining 147 patients were classified under the non-POI group.

### Risk Factors for POI

The defecation time after SIFE was 10 (9–14.75) days in the POI group and 5 (5–6) days in the non-POI group (*P* < 0.001). The univariate logistic regression analysis for POI is shown in Table 1. A total of 11 factors differed between the two groups: the rate of patients with grade V abdominal adhesions, SIFE duration, blood loss during SIFE, the administration of RBC suspension during SIFE, postoperative lactate, postoperative Alb, postoperative Hb, the input of RBC suspension and Alb within 48 h of SIFE, time for recovery of lactate <2 mmol/L, and time for recovery of Alb >30 g/L.

**Table 1:** The univariate logistic regression analysis for POI.

**Clinical variables**	**POI group** **(*n* = 100)**	**non-POI group** **(*n* = 147)**	***p***
Female, *n*, %	46 (46%)	72 (48.98)	0.698
Age, (years; mean ±*SD*)	40.58 ± 9.89	39.68 ± 11.21	0.516
BMI, kg/m2, (median, IQR)	19.5 (18.5–22.75)	20 (19–22.75)	0.771
Length from treitz to location of fistula, cm (median, IQR)	120 (100–200)	140 (100–200)	0.362
Length from location of fistula to ileocecum, cm (median, IQR)	165 (130–245)	150 (120–250)	0.314
Etiology, *n*, %			0.444
Trauma	56 (56)	92 (62.59)	
Mechanical ileus	37 (37)	49 (33.33)	
Mesenteric thrombosis	7 (7)	6 (4.08)	
Operation leading to fistula, *n*, %			0.451
Repair	27 (27)	33 (22.45)	
Excision and anastomosis	73 (73)	114 (77.55)	
Patients with open abdomen or enteroatmospheric fistula	13 (13)	21 (14.29)	0.852
Interval from fistula occurrence to SIFE, days, (mean ±*SD*)	141.12 ± 31.28	134.85 ± 34.22	0.220
Output of fistula, (ml; mean ±*SD*)	375.59 ± 101.99	392.22 ± 121.51	0.262
ASA scores before SIFE (median, IQR)	1 (1–2)	1 (1–2)	0.947
SIFE duration, minutes, (mean ±*SD*)	254.22 ± 50.59	188.88 ± 94.27	**<0.001**
Preoperative Hb, g/L, (mean ±*SD*)	111.21 ± 9.57	112.96 ± 11.24	0.204
Preoperative Alb, g/L, (mean ±*SD*)	36.08 ± 2.01	36.55 ± 2.71	0.140
Length of small intestine after SIFE, cm (median IQR)	240 (200.50–280.75)	260.50 (210–300)	0.094
Blood lose during SIFE, ml, (median IQR)	1859.26 ± 268.11	1194.22 ± 421.09	**<0.001**
The input of RBC suspension during SIFE, U, (median IQR)	5 (4–6)	3 (3–4)	**0.001**
Abdominal adhesion grade^*^, *n* (%)			**<0.001**
IV	41	111	
V	59	36	
Number of anastomoses, *n*, (median, IQR)	2 (2–2)	1 (1–2)	0.097
Postoperative vasoactive drugs, *n* (%)	37 (37)	38 (25.85)	0.061
Postoperative lactate, mmol/L (mean ±*SD*)	6.27 ± 1.87	4.75 ± 2.54	**<0.001**
Postoperative Alb, gl/L (mean ±*SD*)	23.01 ± 0.77	27.29 ± 1.03	**<0.001**
Postoperative Hb, gl/L (mean ±*SD*)	81.12 ± 1.70	88.94 ± 2.56	**<0.001**
The input of RBC suspension within 48 h after SIFE, U, (median IQR)	5.5 (4–6.5)	4 (2–4)	**0.002**
The input of Alb within 48 h after SIFE, g, (median IQR)	60 (60–80)	60 (40–80)	**0.041**
Time for recovery of lactate <2 mmol/L, days; (median IQR)	4 (2–5)	2 (1–3)	**<0.001**
Time for recovery of Alb > 30 g/L, days; (median IQR)	3 (2–3)	2 (1–2)	**<0.001**
Time for recovery of RBC > 100 g/L, days;(median IQR)	2 (1–2)	2 (1–2)	0.299
Comorbidity, *n* (%)			
Hypertensio	12 (12)	20 (13.61)	1.000
Diabetes mellitus	8 (8)	12 (8.16)	1.000

*Alb, albumin; BMI, body mass index; Hb, hemoglobin; SIFE, definitive resection of anastomotic leakage; RBC, red blood cell*.

* *Grade I= no adhesions; grade II= minimal adhesions localized to 1 or 2 areas; grade III=diffuse adhesions, but not extensive; grade IV=diffuse extensive adhesions, easily lysed; grade V=diffuse extensive, dense adhesions, difficult to lyse. Bold values indicate p < 0.05*.

The 11 factors mentioned above were included for a multiple logistic regression analysis. Then, the following four factors were confirmed: blood loss during SIFE (OR = 1.001; 95% CI: 1.000–1.259; *P* = 0.012), postoperative lactate(OR = 1.212; 95% CI: 1.001–1.304; *P* = 0.015), grade V abdominal adhesions (OR = 2.518; 95% CI: 1.814–3.44; *P* = 0.024), and time for recovery of lactate <2 mmol/L (OR = 2.079; 95% CI: 1.599–3.616; *P* =0.026, Table 2).

**Table 2:** The multi-logistic regression analysis for postoperative POI.

**Clinical variables**	**OR**	**95%CI**	***P***
SIFE duration	1.006	0.875–2.011	0.288
Blood lose during SIFE	**1.001**	**1.000–1.259**	**0.012**
The input of RBC suspension during SIFE	2.455	0.771–5.185	0.535
Abdominal adhesion grade^*^, *n* (%)			
IV	Ref	-	-
V	**2.518**	**1.814–3.440**	**0.024**
Postoperative lactate	**1.212**	**1.001–1.304**	**0.015**
Postoperative Alb	0.922	0.781–1.076	0.091
Postoperative Hb	0.995	0.519–1.065	0.187
The input of RBC suspension within 48 h after SIFE	1.675	0.599–2.105	0.391
The input of Alb within 48 h after SIFE	1.054	0.233–3.047	0.759
Time for recovery of lactate <2 mmol/L	**2.079**	**1.599–3.616**	**0.026**
Time for recovery of Alb>30 g/L	1.798	0.876–3.471	0.097

*Alb, albumin; BMI, body mass index; Hb, hemoglobin; SIFE, definitive resection of anastomotic leakage; RBC, red blood cell*.

* *Grade I= no adhesions; grade II= minimal adhesions localized to 1 or 2 areas; grade III=diffuse adhesions, but not extensive; grade IV=diffuse extensive adhesions, easily lysed; grade V=diffuse extensive, dense adhesions, difficult to lyse. Bold values indicate p < 0.05*.

In addition, there were 21 patients with a defecation time more than 20 days after SIFE. The median defecation time of the 21 patients was 27 (24–32) days. After a logistic regression analysis, it was revealed that grade V abdominal adhesions (OR = 7.449; 95% CI: 1.283–15.817; *P* = 0.031), and time for recovery of lactate <2 mmol/L (OR = 2.993; 95% CI: 1.096–6.778; *P* =0.039) were the factors associated with defecation time more than 20 days.

### Outcomes of Patients With POI

No intra-operative deaths were recorded. A total of 71 patients (71/247; 28.74%) had a recurrent fistula. Of the 71 patients, 37 patients were included in the POI group (37/100; 37%), and 34 patients were in the non-POI group (34/147; 23.13%, *P* = 0.018). Of the 71 patients, 16 received an emergency operation or abdominal puncture due to abdominal infection resulting from a recurrent fistula [8 (8%) in the POI group vs. 8 (5.44%) in the non-POI group; *P* = 0.438]. A total of 8 (4.59%) patients died after SIFE [3 (3%) in the POI group vs. 5 (3.40%) in the non-POI group; *P* = 1.000].

No patient received a re-operation due to the POI (intra-intestinal splinting performed and mechanical ileus not considered). Except for the patients who died, all patients with POI received conservative treatment and gradually had return of bowel function.

The postoperative hospital stay was 57.11 ± 50.92 days in the POI group, which was significantly higher than that in the non-POI group (38.56.03 ± 34.91 days, *P* = 0.001). An adjusted cox-regression analysis showed that POI was associated with the prolonged postoperative stay in the hospital (HR = 3.291; 95% CI: 2.511–4.172; *P* = 0.014).

## Discussion

In our study, we investigated the risk factors and outcomes of POI in patients with SIFE. To our knowledge, this might be the first study focused on POI in this special group of population. In this study, we revealed that blood loss during SIFE, grade V abdominal adhesions, postoperative lactate, and time for recovery of lactate <2 mmol/L were the four risk factors of POI in patients with diffuse extensive abdominal adhesions who underwent a SIFE.

Grade V abdominal adhesions leading to extensive manipulation affected the defecation time through the inflammatory response (7). The response always started on the 3rd to 4th h after surgical manipulation (11). The release of pro-inflammatory cytokines and chemokines caused the up-regulation of intracellular adhesions molecules in the endothelium (12). Phagocytes residing throughout the gut were activated, resulting in the migration of leucocytes to the muscularis externa. The release of nitric oxide and prostaglandins by these phagocytes prevented peristalsis by inhibiting smooth muscle contractility directly.

In a previous study, Artinyan et al. (10) revealed that blood loss during the operation was associated with POI in patients with abdominal surgery. In addition to the surgical injury of the intestine, which leads to a local inflammatory reaction in the abdominal cavity, Artinyan et al. argued that increased blood loss could potentially lead to disorders of the neuroendocrine system, a greater traumatic sympathetic and endocrine stress response, inhibiting gastrointestinal transit and leading to POI (10). Despite the neuroendocrine system disorders, compared with routine abdominal surgery, the local abdominal inflammatory response resulting from surgical stress and trauma following blood loss during SIFE might be more severe. In our study, blood loss during SIFE was mainly due to the extensive infiltration of blood between adherent tissues. Intraoperative compression hemostasis was the primary method of extensive infiltration of blood. Long-time compression hemostasis during SIFE and frequent change of the abdominal gauze led to frequent abdominal surgical stress, which led to over-stimulated inflammatory response and caused POI. In addition, intra-operative and postoperative fluid therapy for massive bleeding during SIFE might lead to gastrointestinal edema (10, 13). This might increase the possibility of POI. In summary, to some extent, the blood loss during SIFE could reflect the influence of the local and systemic injuries on the gastrointestinal physiological function.

In our study, we discovered that postoperative lactate was associated with POI. The overproduced and underutilized lactate was regarded as the result of impaired mitochondrial oxidation in patients with tissue hypoxia or ischemia (14). Lactate could be generated by inflammation in relation to the systemic inflammatory response following critical illness (15). Hyperlactatemia can reflect global or regional hypoperfusion. Postoperative hyperlactatemia was associated with increased surgical complications, mortality, and increased length of stay in patients receiving heart surgery, neurosurgery, and abdominal surgery (16–18). The common explanation of postoperative hypoperfusion was that it was due to impairment of microvascular blood flow both before and after major abdominal surgery (19–21). Microvascular blood flow impairment might lead to postoperative systemic or local inflammation response (22, 23), which negatively influences gastrointestinal motility (24, 25). In addition, recently, more and more researchers paid attention to postoperative changes in lactate levels. Creagh-Brown et al. (26) showed that the positive linear relationship between the lactate levels and risk of mortality continued down into the normal range of lactate after abdominal surgery. In addition, Jelena Veličković et al. (19) revealed that the lactate at 12 h after abdominal surgery would predicate the incidence of morbidity most accurately. It was presumed that lactate clearance was closely associated with inflammation (22, 23). Postoperative inflammation would influence the incidence of complications and mortality (24, 25). Our study again confirmed that the time of lactate recovery was associated with postoperative POI. It has also revealed that the time of lactate recovery was also a risk factor for defecation time more than 20 days.

Our study had the following limitations. First, as the study was retrospective, the selection bias existed. Second, the sample size of our study was small, which led to bias as well. Third, accurate statistics of postoperative fluid input and output are missing, which might influence POI. In addition, our postoperative fluid management was traditional, and we did not use intra- and postoperative goal-directed fluid therapy in our study. In other words, our fluid management was primarily based on the changes in HR, CVP, and MAP. However, according to contemporary medicine, the HR, CVP, and MAP were unreliable indicators of volume status. While adequate fluid resuscitation is beneficial to lactate clearance, the excessive liquid loading might lead to tissue edema, and the intestinal edema could lead to a longer defecation time.

In our experience, the grade of abdominal adhesions could not be changed before SIFE. Intraoperative bleeding is often extensive and is usually due to dissociated adhesions; bleeding management is also very difficult. However, as we have discussed above, our intraoperative and postoperative rehydration strategy is relatively rough. It is difficult to say whether there is a relative lack or excess of rehydration. As a result, further study could be focused on the relationship between perioperative fluid replacement and lactate. In addition, whether the drugs improving microcirculation could reduce postoperative lactate and improve postoperative POI might be the further research direction. From our research, we can see that the incidence of POI was very high after SIFE in patients with diffuse extensive abdominal adhesions. In our opinion, if grade V abdominal adhesions were present or massive bleeding was detected intraoperatively, or hyperlactemia due to intraoperative shock was detected postoperatively, we should expect POI, and occasionally defecation time as long as a month. Furthermore, empirically speaking, this group of patients would have more serious abdominal adhesions after SIFE. We still need to be careful of mechanical POI caused by intestinal angulation caused by abdominal adhesions after SIFE. Our method was intra-intestinal splinting, which seemed to be effective. No patients received re-operation due to the mechanical POI. Of course, the most important thing is to wait patiently for defecation.

## Conclusion

In patients with diffuse extensive abdominal adhesions, the blood loss during operation, grade V abdominal adhesions, and time for recovery of lactate <2 mmol/L were risk factors for POI after SIFE. The duration of POI was relatively long. However, with intra-intestinal splinting during SIFE, an emergency reoperation has been avoided.

## Data Availability Statement

The raw data supporting the conclusions of this article will be made available by the authors, without undue reservation.

## Ethics Statement

The studies involving human participants were reviewed and approved by NJZY-20200023. The patients/participants provided their written informed consent to participate in this study.

## Author Contributions

XX and ZY collected and analyzed the data. WT, MY, and XX wrote the main manuscript text. XX prepared figures. RZ and ZY designed the research. All authors contributed to the article and approved the submitted version.

## Conflict of Interest

The authors declare that the research was conducted in the absence of any commercial or financial relationships that could be construed as a potential conflict of interest.
